# Wealth in Couples: Introduction to the Special Issue

**DOI:** 10.1007/s10680-022-09640-5

**Published:** 2022-09-22

**Authors:** Philipp M. Lersch, Emanuela Struffolino, Agnese Vitali

**Affiliations:** 1 Humboldt-Universität zu Berlin, Berlin, Germany; 2 DIW Berlin/SOEP, Berlin, Germany; 3 University of Milan, Milan, Italy; 4 University of Trento, Trento, Italy

**Keywords:** Wealth, Couples, Life course, Gender, Inequality, Partners

## Abstract

The assumption that economic resources are equally shared within households has been found to be untenable for income but is still often upheld for wealth. In this introduction to the special issue “Wealth in Couples”, we argue that within-household inequality in wealth is a pertinent and under-researched area that is ripe for development. To this end, we outline the relevance of wealth for demographic research, making the distinction between individual and household wealth. Drawing on a life-course perspective, we discuss individual wealth accumulation within couples and its links to family-demographic processes, the institutional context, and norms on pooling and sharing. We conclude with a brief summary of the main findings from the special issue and highlight implications for demographic research and for future research in this field.

## Introduction

Since the 1960s, women’s access to employment opportunities and economic resources has improved their autonomy and, relatedly, their bargaining power within the household. These dynamics are accompanied by an unprecedented value shift towards individualism, autonomy, and personal fulfilment known as the “Second Demographic transition” (Lesthaeghe, 2020), a change in the meaning of marriage, which has become increasingly “individualised” (Cherlin, 2004), and the emergence of family formation patterns alternative to marriage. All of these transformations affected how economic resources are treated by partners within couples.

The empirical evidence shows that partners are increasingly more likely to keep and manage at least part of their economic resources separately, which means that partners within couples may have unequal access to the household’s monetary resources (e.g. Bennett, 2013; Frémeaux & Leturcq, 2020; Sauer et al., 2021). This contradicts the idea of the unitary household model, which considers the household as one economic unit with the implicit expectation that economic resources such as income and wealth are equally shared and jointly accumulated among partners. The unitary household model ignores potentially persistent gender differences in the accumulation of economic resources within households.

Against the background of high and increasing wealth inequality in rich countries (Chancel et al., 2021), a stream of literature on within-household inequality in wealth has emerged (see, e.g. Frémeaux & Leturcq, 2020; Nutz & Lersch, 2021; Lersch, 2017b). This literature indicates that money can be jointly or separately saved and invested by partners in couples, wealth can be transferred between partners, and individuals may restrict their partners’ access to personal wealth, all affecting individuals’ wealth accumulation and economic well-being. The present special issue “Wealth in Couples” extends this nascent literature by examining inequality in *individual wealth* accumulation of women and men in opposite-sex couples as a function of both institutional and household contexts. Additionally, it reflects on the consequences of demographic processes for these inequalities and the consequences of inequality for demographic behaviour. As union dissolution, re-partnering, and complex families become more common, scholars must devote attention to individual—rather than only household—wealth accumulation in order to identify how women and men may be differentially exposed to the economic consequences of important life-course transitions such as separation, widowhood, retirement, and unemployment.

The special issue consists of five original, empirical studies that complement each other in pursuing two innovative contributions: First, they shed light on the existence of *within-couple wealth inequalities* by documenting the gender wealth gap among partners, using various unique datasets on net wealth, pension rights, and matrimonial property regimes.^1^ Second, the studies identify *factors affecting within-couple wealth inequalities* by highlighting the role of demographic processes as well as institutional contexts in shaping these inequalities. Demographic processes (union formation, union dissolution, and parenthood), the division of paid and unpaid labour within households, and gendered social norms and practices are likely to affect couples’ wealth management. The national institutional context provides the environment for individuals’ wealth accumulation within couples. For instance, matrimonial property regimes and statutory pension schemes differ across countries and affect wealth accumulation by determining the extent to which individuals in different couple types (such as marriages, non-marital cohabitations, and registered partnerships) are able to participate in and benefit from their partners’ financial resources. Finally, the present contributions analyse the link between wealth ownership structure in the households and gender *differences in subjective wellbeing*.

All in all, each article of this special issue addresses more than one open question in this emerging literature. We will discuss those questions in the following sections. The contributions to this special issue are not just relevant because they make theoretical and empirical advances but also because they can inform policy and institutional design. The analysis of how differences in (the accumulation of) economic resources such as wealth are associated with demographic processes is crucial to determine to what extent policies redistribute wealth among individuals rather than households and the implications in terms of intergenerational transmission of disadvantage and persistent gender differences in future generations. This is even more relevant, given that demographic behaviours such as union dissolution and single parenthood have an educational gradient. Education is one of the factors associated with access to wealth and the potential to accumulate wealth (Pfeffer & Killewald, 2018). For example, low educated individuals may be more likely to experience single parenthood, and single parents (and especially single mothers) may have limited or no wealth to rely upon and little opportunity to save and invest for their and their children’s future.

In the next section, we outline the relevance of wealth for demographic research. We first characterise wealth as a dimension of inequality to be considered over and above income. Going beyond the unitary household model allows us to look at gender differences in the accumulation of resources over the life course and to better interpret the relationship between wealth and demographic processes. Section 3 discusses individual wealth accumulation within couples and incorporates arguments and findings from the contributions to the special issue. Here, the life-course perspective is pivotal as it offers conceptual tools to analyse the wealth accumulation process and its link to family-demographic processes in the light of intergenerational dynamics, the institutional context (e.g. tax and pension systems, housing and matrimonial property regimes), and norms on pooling and sharing. Section 4 concludes with a brief summary of the main findings from the articles in this special issue by highlighting their implications for future research in the field of demography and beyond.

## The Relevance of Wealth in Demography

### Wealth in General

Wealth (or net worth) is a stock of economic resources and provides consumption potential. It is usually defined as the sum of the value of all privately owned assets of a household or an individual minus debts (Killewald et al., 2017). Assets include self-occupied homes and other real estate, financial assets, vehicles, life insurance, private pension plans, business assets, and tangible assets such as jewellery and artworks. From these assets, all outstanding debts and loans, including mortgages, credit cards, and student loans, are subtracted. This means that households and individuals may have negative net wealth. For most households and individuals, their self-occupied homes are the most significant wealth component in their portfolios. The inclusion of public pension wealth has been debated in the literature (Cordova et al., 2022, this issue).

(Labour) incomes are a significant source of wealth, but wealth as a dimension of social inequality is distinct from income, and income and wealth are only moderately correlated (Killewald et al., 2017). Unlike income flows, wealth represents a stock of economic resources that is accumulated or consumed over a considerable amount of time—even over centuries, when considering intergenerational transfers. Wealth provides advantages that go beyond labour income—it provides benefits like housing for homeowners as well as rent and may be liquefied to generate income. Moreover, wealth provides a safety net for rainy days as well as power and status and can be transferred to the next generation (Keister & Moller, 2000; Spilerman, 2000). Finally, wealth and its returns are often treated more advantageously in tax systems than labour income.

In recent years, there has been a burgeoning interest in wealth (Piketty, 2014). It is increasingly acknowledged that wealth inequality is immense in many rich democracies, with about twice as much wealth inequality as income inequality in most OECD countries (Balestra & Tonkin, 2018; Chancel et al., 2021). In retrenching welfare states, private wealth may be increasingly important for maintaining standards of living. Likewise, for people in pension systems facing demographic challenges, the re-marketisation of the provision has made it even more important to accumulate private wealth (Ebbinghaus, 2015). There is also mounting evidence that the intergenerational transmission of wealth is (increasingly) contributing to the persistence of advantages and disadvantages in the next generation (Dräger, 2021; Hällsten & Pfeffer, 2017; Pfeffer, 2018).

The growing scholarly attention to wealth can also be attributed to improvements in (longitudinal) data availability in recent years, including large-scale cross-national surveys (see Killewald et al. (2017) for an overview of surveys with wealth measurements). However, a remaining issue for research is the fact that most surveys record wealth at the household level rather than at the individual level. As discussed in the next section, this limits researchers in addressing demographic research questions. However, improvements in this regard have taken place in recent years, and—to the best of our knowledge—individual-level wealth can now be examined to varying degrees in seven large-scale surveys:^2^ the British Household Panel Study and its successor Understanding Society—UK Household Longitudinal Study, the UK Wealth and Assets Survey (from 2018), the European Household Finance and Consumption Survey (for selected countries including Austria and France), the Household, Income and Labour Dynamics in Australia Survey, the US Survey of Income and Program Participation, and the German Socio-Economic Panel Survey. The Socio-Economic Panel Survey provides the most comprehensive measurement of individual-level wealth.

### Individual Wealth Versus Household Wealth

Wealth is often considered a characteristic of households and families (Spilerman, 2000) in line with unitary household models in new home economics theory (Becker, 1981; Samuelson, 1956). This theory proposes that all household economic resources are pooled to maximise a joint household utility function. Yet, in their review of research on the intra-household sharing of economic resources, Kulic and Dotti Sani (2020) have highlighted how the sociological literature has long departed from the unitary model governing family decisions first theorised by Samuelson in 1956, a period characterised by the dominance of the male-breadwinner female-homemaker married couple.

Sociologists Blood and Wolfe (1960) proposed the so-called resource theory, which is an alternative to the unitary model, predicting that economic resources within couples are allocated according to the partners’ relative resources: the partner with the highest economic resources has more weight in the bargaining process. It follows that when couples have similar economic resources, they may be more inclined to pool them, whereby in couples with an imbalance in economic resources, these are more likely kept separate (see, e.g. Fraboni & Vitali, 2019). The economic literature recognised the importance of bargaining power as shown, for example, by game-theoretic (Lundberg & Pollak, 1996) and independent decision-making models (Grossbard, 2011). Bargaining power hence appears to be an important factor for explaining intra-household allocation of economic resources.

Empirical research has found evidence against the unitary model of resource sharing, demonstrating a trend within partnerships towards keeping at least some income separate (Pahl, 1989). These studies mostly focused on income sharing, but similarly there is growing evidence that unitary models are not adequate for describing how wealth is shared within couples (Deere & Doss, 2006; Joseph & Rowlingson, 2012). Therefore, household-level measures may not accurately capture individuals’ financial well-being within households (Kapelle et al., 2022, this issue; Lersch, 2017a; Tisch, 2021), because individuals do not equally share wealth within the household.

When studying wealth within households, it is important to distinguish between the ownership (legal property rights) and the actual control of assets (Tisch & Lersch, 2021). Ownership of assets will depend on the institutional context within which marital property regimes and other legal rules are relevant, as we discuss below (Deere & Doss, 2006; Fraboni & Vitali, 2019). Regarding property rights, it is relevant to differentiate between solely held wealth (i.e. wealth held only in one partner’s name) and jointly held wealth (i.e. wealth held in both partners’ names) (Frémeaux & Leturcq, 2018, 2020). Kapelle et al. (2022, this issue) argue that jointly held wealth likely reflects norms of sharing responsibilities and resources within marriage, while solely held wealth may offer individual economic independence. Irrespective of property rights, individuals may unequally control certain assets, that is, they may have unequal leverage in making decisions about their use. While the vast literature on financial management in couples suggests some explanations for unequal control, these explanations have not been fully applied in wealth research yet (Joseph & Rowlingson, 2012; Tisch & Lersch, 2021).

As mentioned above, until recently, a lack of data made it difficult to systematically account for the separation of wealth between partners (Doss et al., 2020). However, the increasing availability of information on individual wealth of all adults in the household made it possible to go beyond the study of gender inequality in wealth based on single-person households (Schneebaum et al., 2018). Previous literature for Germany found an average gender wealth gap in favour of men of about EUR 30,000 (Sierminska et al., 2010). Within couples, the gender gap increased to EUR 33,000, with a more significant gap in couples with children under the age of 16 years (EUR 36,000) than in childless couples (EUR 20,000) (Grabka et al., 2015). The gender gap in couples is most significant in business assets, insurance, and private pension assets, while housing assets are more equally distributed between women and men. Between 2002 and 2012, the gender wealth gap in Germany decreased at the bottom and top of the wealth distribution, while it stagnated at the median (Sierminska et al., 2019). Similar evidence regarding the general gender wealth gap has been found in other diverse contexts, for example, in Ecuador (Anglade et al., 2017), Estonia (Meriküll et al., 2021), France (Frémeaux & Leturcq, 2020), and the USA (Lee, 2022; Sariscsany, 2020).

The gender wealth gap differs across subgroups, and one important dimension of heterogeneity is by migration status and race, for example, through structural disadvantages in the labour market and the consequences of historical exploitation persisting across generations. Rehm et al. in this special issue (2022) show that the gender wealth gap is highest in couples with a foreign woman and a native man in Austria (i.e. foreign women hold considerably less wealth than their male partners). Previous findings for Italy, however, show that couples with a foreign and a native spouse are more likely to choose community of marital property than native couples, hence ‘protecting’ the foreign spouse with equal sharing of marital wealth in case of divorce, independently of his/her gender (Fraboni & Vitali, 2019). Lee (2022) finds the gender wealth gap to be more pronounced among the non-Whites compared to Whites in the USA. The intersectionality of, one the one hand, gender and, one the other hand, migration status and race in individual-level wealth inequalities is an important area for further exploration.

## Individual Wealth Accumulation in Couples from a Life-Course Perspective

The life-course perspective offers analytical tools to conceptualise wealth accumulation within couples in terms of: (1) individual and household wealth accumulation along the life course, both within a given generation and across generations (life-span development); (2) the link between the wealth accumulation process and family demographic processes, such as union formation and union dissolution (multidimensionality of the life course); (3) the role of socio-historically and geographically identified institutional contexts (time and place); (iv) norms around pooling and sharing (relationship between individual agency and structure) and their implications for the individual and their partner’s well-being (linked lives).

### Intra- and Intergenerational Dynamics

During individuals’ lives, wealth—at the household and personal level—mainly grows out of surplus income (i.e. incomes minus consumption) and wealth transfers, which link wealth accumulation processes across generations (Frémeaux & Leturcq, 2022, this issue). Investment strategies, as well as structural opportunities for and constraints on investments, determine how wealth grows. The consumption of assets, loss-making investments, and outgoing transfers reduce wealth (Chang, 2010). Wealth generates further wealth when it appreciates, when it can be used as collateral for additional investments, and when its returns can be reinvested. Because of these characteristics, the dynamics of wealth inequalities are used as an example of cumulative (dis-) advantage (CAD) processes (Dannefer, 1987 & O’Rand, 1996).

Intergenerational wealth transfers are a significant source of wealth and extend the accumulation of wealth beyond individuals’ life courses (Gale & Scholz, 1994). The relative importance of transfers compared to savings from surplus income for wealth accumulation is empirically controversial, but recent studies show that inherited wealth likely represents around 50–60% of total wealth (Alvaredo et al., 2017) and that those in the middle of the wealth distribution are likely to benefit more from transfers in terms of improvements in their wealth position than those further down or up in the distribution (Korom, 2018). Moreover, the relative importance of transfers and income depends on the life-course phase, since large capital transfers from parents often only occur in the second half of the adult child’s life (Pfeffer & Killewald, 2018).

Regarding intergenerational wealth transfers, the literature has not, for the most part, found systematic gender differences (Cox, 2003; but see also Bessière, 2019). However, wealth transfers play a more critical role in wealth accumulation by women than men, as women gain less personal wealth from earnings due to their typically disadvantaged labour market positions (Deere & Doss, 2006). Wealth transfers may have substantial effects on the within-couple wealth gap: for example, the wealth gap between partners is wider and tends to favour men if men inherit wealth, while the gap is smaller if women inherit wealth (Grabka et al., 2015).

For both men and women, the potential for wealth accumulation is directly linked to their employment and earnings (Cordova et al., 2022, this issue). Women may discontinue or reduce their labour force participation after childbirth, while men generally continue to work full time. When employed, women earn less than men, on average, and such structurally disadvantaged labour market positions can mean they have a lower capacity to accumulate wealth, including pension entitlements (Grabka et al., 2015; Madero-Cabib & Fasang, 2015; Ruel & Hauser, 2013). Furthermore, evidence shows that women’s lower likelihood of being self-employed and their higher likelihood of working in sociocultural professions contribute to explaining the gender wealth gap beyond simple earning differences (Waitkus & Minkus, 2021). Finally, Chang (2010) has used the metaphor of the “wealth escalator” to describe how multiple advantages for men (for example, fringe and tax benefits) additionally widen the gender wealth gap beyond earnings.

### Wealth in Couples and Family-Demographic Processes

The process of accumulation of wealth in couples is linked to family-demographic processes: fertility, union formation, and union dissolution. In what follows, we provide an overview of research and outline possible mechanisms.

The relationship between wealth and fertility has been studied from various angles. First, household wealth gives economic stability for forming a family and positively influences fertility (Hackman & Hruschka, 2020). For instance, increases in households’ housing wealth among homeowners are positively related to both intended and realised fertility, for example because self-occupied homes are considered superior environments for childrearing (Atalay et al., 2021; Clark & Ferrer, 2019; Lovenheim, 2011). Traditionally, entry into homeownership and fertility have been closely linked events, but this relationship depends on housing market characteristics (Mulder & Wagner, 1998). Furthermore, recent housing market dynamics that have made homeownership less affordable may weaken the relationship (Tocchioni et al., 2021). Finally, household wealth has been found to be negatively related to unintended fertility (Su & Addo, 2018): this relationship is likely to be mediated by socio-economic background.

Second, fertility influences wealth. On the one hand, the higher the number of children, the higher the household consumption and hence the lower the savings and possibility to accumulate wealth. On the other hand, children may increase the preferences for saving and entering homeownership (Lersch et al., 2017). Potentially as a result of these counteracting factors, several studies suggest a curvilinear association, where having up to three children is positively associated with household wealth but more than three children is negatively associated with household wealth (Vespa &, Painter, 2011; Zissimopoulos et al., 2015). More research is needed to understand how and why this nonlinear association emerges by considering not just the resources actually available to the household to “afford” raising three or more children, but also preferences, which might differ, for example, between low- and highly educated couples.

Moreover, the timing of fertility may matter for wealth accumulation: early parenthood influences parents’—especially women’s—employment and earnings trajectories, again influencing the ability to accumulate wealth. Independently of the timing of first birth, parenthood contributes to gender inequalities in wealth, with mothers being more disadvantaged in their personal wealth accumulation than fathers (Lersch et al., 2017). Following a birth, the employment and earning trajectories of mothers differ considerably from those of fathers, resulting in considerable declines in the mother’s share of couple earnings following first birth, declines that persist over several years and that is particularly visible among lower-educated mothers (Musick et al., 2020).

Third, fertility in the family of origin is linked to wealth accumulation later in life: an adult’s number of siblings has been found to be negatively associated with wealth, because resources from parents are diluted with more siblings around. Relatedly, changes in fertility dynamics and the persistently low and lowest-low fertility (i.e. total fertility rate below 1.3) characterising several high-income countries may have a direct impact on future inheritances. Finally, there is also evidence that birth order remains a significant predictor of wealth even in contemporary societies, i.e. first-borns tend to have more access to wealth compared to second- and higher-order siblings (Killewald, 2013; Lersch, 2019).

Household wealth, just like income, stable employment, and economic resources more generally, has been found to facilitate union formation, specifically entry into marriage (Schneider, 2011), which is, vice versa, delayed by debt and loans (Addo, 2014). At the same time, household wealth increases partnership stability for cohabiting and married couples, while unsecured debt may increase instability (Eads & Tach, 2016; Lersch & Vidal, 2014). Eads and Tach (2016), one of the few studies considering the consequences of within-household wealth inequality on unions, found that union stability is lower among couples where only one of the partners owns the home compared to both partners owning the home (see also Eads et al., 2022).

The question of whether cohabitation is associated with changes in wealth levels is contested (Lersch, 2017b; Ozawa & Lee, 2006). Marriage is positively related to household and personal wealth for women and men and does not further increase within-couple inequality in personal wealth (Kapelle & Lersch, 2020; Lersch, 2017b). Frémeaux and Leturcq (2022, this issue) show that the marital property regime chosen upon marriage is also associated with wealth accumulation: married couples with a separate property regime accumulate more wealth than couples with a community property regime. The dissolution of cohabitation and marriage is associated with significant wealth losses and with the exit from homeownership; in this context, women are mostly—conditional on the institutional context—more disadvantaged than men (Boertien & Lersch, 2021; Kapelle & Baxter, 2021; Lersch & Vidal, 2014).

As described above, union formation and fertility are relevant for wealth accumulation and may have gender-specific outcomes that are relevant for within-couple inequalities. For instance, men are likely to enter unions with more wealth than their female partners, as men are older on average, hence entered in the labour market and started earning and saving earlier compared to women (Sierminska et al., 2010). Marriage between natives and migrants can also directly impact within-couple inequality (Rehm et al., 2022, this issue). Additionally, how personal wealth influences partner choices is crucial: previous research has found positive assortative mating related to personal and parental wealth (Charles et al., 2013; Fagereng et al., 2022). Later union formation may be related to having more separately held wealth as both partners may have accumulated wealth before forming a partnership. Once a union has been formed, remaining in a union increases the overall household wealth. While marriage is mostly associated with joint wealth, previous studies show that during cohabitation, many partners retain individually held wealth (Joseph & Rowlingson, 2012). After experiencing separation following a first cohabitation or divorce, partners are more likely to opt for an unequal distribution of assets in their higher-order unions, especially if they have children from previous unions (Joseph & Rowlingson, 2012; Zagorsky, 2005).

### Institutional Context

Family law differs across countries and over time and has important implications for wealth differences within couples (Deere et al., 2013). For married couples, the marital property regime is decisive. In most European countries, married couples and—in countries where they are available, such as France—registered partnerships, benefit from more legal rights compared to cohabiting couples (Miho & Thévenon, 2020; Perelli-Harris & Gassen, 2012). In addition, laws concerning inheritance and transfer regimes are essential for wealth inequalities. A global trend toward gender equity in inheritance laws has created more equality in inheritances (Deere & Doss, 2006). Table 1 offers an illustrative and selective overview of the institutional context regarding property regimes, pensions, taxation systems, and the housing markets of the countries-cases included in the special issue.

**Table 1:** Overview of institutional context in countries included in the special issue

Country	Institutional dimension								
	Default marital property regime^a^	Pension system		Tax system^d^			Housing market^e^		
		Net pension replacement rate (men)^b^	Gender pension gap^c^	Joint filing	Income splitting	Dependent spouse allowance	Marketisation of housing^g^	Relevance of familial resources	Alternatives outside of homeownership sector
Austria	Separate property	87.1	35.0	No	No	Yes	High	Med	Many
France	Community property	74.4	29.4	Family tax splitting		No	High	Low	Many
Germany	Community of accrued gains	52.9	32.7	Yes	Yes	No	High	Low	Many
Italy	Community property^f^	81.7	34.9	No	No	Yes	Medium	High	Few

^a^http://www.coupleseurope.eu/ (last accessed 15/03/22)

^b^https://data.oecd.org/pension/net-pension-replacement-rates.htm#indicator-chart (last accessed 15/03/22)

^c^https://ec.europa.eu/eurostat/databrowser/view/ilc_pnp13/default/table?lang=en (last accessed 15/03/22)

^d^Schechtl (2021)

^e^Lersch and Dewilde (2015)

^f^Mandatory choice between a separate property and community property regime

^g^Marketisation of housing is high if the mortgage system is well developed, resulting in a more marketised provision of homeownership

The determinants of the choice of property regimes are complex, as are their consequences. For instance, Vitali and Fraboni (2022, this issue) show that among Italian married couples, those with a prior experience of cohabitation differ in the ways they pool marital wealth from those who marry without cohabitation. These differences are found to be more compositional than behavioural. Leturcq and Frémeaux (2022, this issue) observe that, in France, couples in registered partnerships (PACS) who choose a separate property regime tend to accumulate more wealth than those in registered partnerships who choose a community property regime. Furthermore, Leturcq and Frémeaux find that the woman’s share of household wealth increases over time for couples in a separate property regime but not for couples in a community property regime.

The welfare system has a substantial influence on these inequalities and on normative expectations for women’s labour force participation (Ostner & Lewis, 1995). Moreover, the welfare system has direct effects on wealth accumulation. This is most obvious for the accumulation of public and private pension wealth, which is a specific but highly relevant type of wealth (Bönke et al., 2020; Cordova et al., 2022, this issue). Pension systems structure the consequences of gendered life courses for the accumulation of pension wealth, for example, through rules regarding care credits (Madero-Cabib & Fasang, 2015). Several other characteristics of the welfare state may be relevant for the accumulation of personal wealth: all else being equal, the incentives and need for private wealth accumulation are higher in lean welfare states. More generally, welfare states can incentivise wealth accumulation—for example, through matched saving plans. Previous research has not identified systematic cross-country variation in wealth accumulation linked to welfare systems, and more work in this direction is needed (Pfeffer & Waitkus, 2021).

Income tax systems may affect wealth inequalities within couples and contribute to unequal wealth accumulation potential. For example, in the UK, individual taxation and tax benefits facilitate separate savings within couples (Kan & Laurie, 2014). In contrast, joint taxation of both partners, as in Germany or the USA, may have the opposite effect. LaLumia (2008) shows that the introduction of joint taxation in some US states in 1948 reduced married women's non-labour incomes significantly. This indicates that women were more likely to individually hold assets in the separate taxation regime than in the joint taxation regime. In addition, tax regimes may have effects on labour supply by women and men in interaction with other contextual conditions, thereby contributing to unequal labour earnings (Dingeldey, 2001).

Homeownership is the largest investment for most individuals and is often only feasible if partners jointly enter homeownership. Thus, homes are mostly jointly owned (Lersch & Vidal, 2016), and there is less within-couple inequality in housing wealth than in other components of wealth (Grabka et al., 2015). Hence, housing markets can have direct consequences for the within-household distribution of wealth. In housing markets with high homeownership rates—such as Spain, Greece, Finland, and Norway (Pfeffer & Waitkus, 2021)—we might expect partners to have jointly held wealth and, thus, less inequality within households. In contrast, in housing markets in which rental accommodation is more widespread, the potential for within-household inequalities may be larger. In addition, ease of access to homeownership will also affect re-entry into homeownership after union dissolution and can thereby affect the distribution of wealth in higher-order unions.

### Norms

The accumulation of personal wealth and within-household inequality depends on norms about individual ownership and property on the one hand and pooling and sharing in couples on the other. While the meaning of marriage shifted from institutional marriage in the nineteenth to individualised marriage in the twenty-first century, with the emphasis being on spouses’ individual choices and independence (Cherlin, 2004), marriage remains a public commitment to a joint, long-term future together (Holland, 2013; Nutz et al., 2022). In the light of these factors, individualised marriage can be expected to result in stronger preferences for keeping money separate and a reduced need for economic support within marriage (Yodanis & Lauer, 2014). On the other hand, the norm of sharing in marriage remains strong (Pepin, 2019; Tisch & Lersch, 2021). Many married couples still opt for (at least partial) pooling of their income and wealth (Nutz & Lersch, 2021; Yodanis & Lauer, 2014). In this regard, holding wealth jointly is explicitly part of the expectations held for married men and women (Dew, 2016). Married people are expected to make joint investments for their future. However, normative expectations about pooling and sharing are less strong among cohabiting couples (Burgoyne & Sonnenberg, 2009). The importance of these norms varies across countries. For instance, in Germany, the norm of sharing is strong but does not entirely override the norm of individually earned income as is the case in Spain (Ludwig-Mayerhofer et al., 2011).

## Conclusion

This introduction to the special issue “Wealth in Couples” has highlighted the relevance of the distinction between individual and household wealth for demographic research and framed the study of wealth in couples by using the analytical concepts and tools offered by the life-course perspective. The five empirical studies included in this special issue make several contributions to wealth studies with respect to the division and sharing of wealth within couples. Besides shedding light on the existence of the within-couple wealth inequalities documented by gender wealth gaps between partners, the studies in this special issue identify factors affecting within-couple wealth inequalities and their consequences for life outcomes considering the role of demographic processes and of the institutional context.

This special issue continues recent efforts to evaluate the consequences of the increasing trend toward separation of economic resources among partners. This trend was observed previously for income—the special issue offers deeper insights into it by considering wealth. By keeping resources separate, individuals can avoid possible conflicts over the allocation of joint resources, protect their wealth from an abusive partner, and exercise freedom in administering their personal resources. Separate resources, hence, go hand in hand with the diffusion of individualised marriage, with the diffusion of less committed unions such as non-marital cohabitation, and overall, with the diffusion of post-materialistic values as predicted by the second demographic transition. On the other hand, separate resources may foster existing inequalities among partners. For example, there are well-known gender wealth gaps to the detriment of women. By adopting a separate-resources approach, non-working women, especially mothers, may have more limited access to economic resources.

The aforementioned trends towards the separation of economic resources among partners have evolved within contexts characterised by different legal statuses beyond marriage, different property regimes, and different pension rights, all of which may affect the gender wealth gap. With respect to legal statuses and property regimes, Fremeaux and Leturcq (2022, this issue) show that, in France, married couples with a separate property regime accumulate wealth faster than other types of couples. Notwithstanding some self-selection of couples into a certain legal status, differences in wealth accumulation remain after accounting for observed characteristics. In the case of Italy, the drivers of the choice between separation or joint property regimes among married couples who either cohabit or not before marriage (Vitali & Fraboni, 2022, this issue) seem to be related to selection mechanisms. With respect to the role of statutory public and occupational pensions for the gender gap in pension wealth, Cordova and colleagues (2022, this issue) show that the large gap in Germany is reduced when accounting for redistributive elements in pension wealth coming from the statutory pension scheme and especially so for individuals with low wealth.

Finally, couples’ wealth ownership structures may have implications beyond gender inequalities in terms of economic outcomes depending on the prevalent norms around marital sharing. Kapelle and colleagues (2022) in this special issue show that the subjective well-being associated with marital sharing of wealth seems to exceed that of economic independence and financial autonomy from the accumulation of solely held wealth for both men and women. This indicates that fulfilling norms of marital jointness by accumulating wealth jointly has positive effects for both partners.

The empirical findings of the studies included in this special issue hint at several avenues for future research. First, research should investigate whether a separation or pooling of economic resources in couples is best for partners in terms of various (subjective) well-being outcomes, and under what conditions. The limited attention devoted to different marital statuses in previous literature is surprising. Studies in this special issue have shown that wealth pooling and wealth accumulation differs considerably across different marital statuses (married couples, cohabiting couples, and, where legal, registered partnerships). Important research questions concern the conditions under which the separation of wealth is a benefit or a loss and the types of couples or individuals who would benefit or lose. It is also important to understand whether there are long-term effects associated with pooling or separating wealth and economic resources more generally, effects that likely intertwine with family and employment-related life courses. In other words, is separate wealth good or bad for women and men later in life, and how does the answer depend on individuals’ socio-demographic characteristics and life courses (e.g. experience of separation or divorce, presence or absence of own children, experience of career interruptions, ill health, etc.)?

Further, it is important to study how wealth is distributed among same-sex couples to identify possible disadvantages at the intersection of legal status and property regimes in contexts that discriminate more or less explicitly against same-sex couples in terms of legal entitlement and against LGBTIQ individuals in terms of opportunities and rewards in the labour market. Finally, the findings of the studies in this special issue leave open the possibility that selection into a partnership status is also based on unobserved characteristics such as risk aversion or gender attitudes. The fact that these dimensions are rarely included in large-scale surveys makes it more difficult to analyse the various mechanisms that might explain not just selection into a given legal status but also the choice of certain property regimes.

Thus, necessary advances in the field are conditional on the availability of data on individual wealth in general and, more specifically, on longitudinal panel data addressing aspects such as risk aversion. In addition, although a few countries have started to collect individual wealth data, it will be necessary to have data on a broader range of countries to identify and test systematic differences in gender inequality in individual wealth and in the link between demographic processes and wealth division in couples as well as its consequences for different outcomes as a function of the institutional context.

## Footnotes

1 While the data used in the articles included in this special issue represent an important step forward in the ability to track individual-level wealth dynamics within heterosexual couples, they are limited in the possibility to study these processes among same-sex couples, in most cases due to the small sample size.

2 Private pension wealth is available at the individual level in more surveys.
